# Visual and Ocular Characteristics of Anisometropic Children

**DOI:** 10.18502/jovr.v19i2.12413

**Published:** 2024-06-21

**Authors:** Zhale Rajavi, Narges Behradfar, Marzieh Sharahi Dizabadi, Bahareh Kheiri, Kourosh Sheibani, Hamideh Sabbaghi

**Affiliations:** ^1^Negah Aref Ophthalmic Research Center, Shahid Beheshti University of Medical Sciences, Tehran, Iran; ^2^Ophthalmic Epidemiology Research Center, Research Institute for Ophthalmology and Vision Science, Shahid Beheshti University of Medical Sciences, Tehran, Iran; ^3^Department of Ophthalmology, School of Medicine, Shahid Beheshti University of Medical Sciences, Tehran, Iran; ^4^Department of Optometry, School of Rehabilitation, Shahid Beheshti University of Medical Sciences, Tehran, Iran; ^5^Ophthalmic Research Center, Research Institute for Ophthalmology and Vision Science, Shahid Beheshti University of Medical Sciences, Tehran, Iran; ^6^Department of Epidemiology and Biostatistics, School of Medicine and Dentistry, Western University, Ontario, Canada; ^7^Basir Eye Health Research Center, Iran University of Medical Sciences, Tehran, Iran; ^9^Zhale Rajavi: https://orcid.org/0000-0002-4078-3017; ^10^Hamideh Sabbaghi: https://orcid.org/0000-0002-2627-7222

**Keywords:** Anisometropia, Demographic Factors, Hyperopia, Myopia

## Abstract

**Purpose:**

To compare the demographic and ocular characteristics of patients with low and high levels of anisometropia compared with non-anisometropic individuals.

**Methods:**

This cross-sectional study was conducted on 1803 individuals (age range, 1 to 30 years) examined at strabismus clinics between January 2019 and December 2020. Of these, 203 subjects had anisometropia (11.2%); 66 cases were excluded due to the history of prior ocular surgery except from strabismus surgery. Finally, data from 137 subjects were analyzed. Spherical or cylindrical differences of 1.50 or 3.00D between the two eyes were defined as low or high anisometropia, respectively, and isometropic subjects (*n* = 1600) served as controls.

**Results:**

No significant difference was observed between cases and controls regarding age (10.25 ± 8.41 vs. 9.2 ± 1.7 years; *P* = 0.133) and sex (*P* = 0.051). History of ocular surgery was present in 33% of anisometropic patients versus 0.8 % of isometropic cases. The rate of amblyopia was 83% and 2.3% in anisometropic and non-anisometropic groups, respectively. Best corrected visual acuity (BCVA) was comparable in amblyopic eyes in both study groups, while BCVA of non-amblyopic eyes of non-anisometropic subjects was better (non-anisometropic: 0.01 ± 0.01 vs. anisometropic: 0.06 ± 0.17 LogMAR; *P* = 0.001). Eye deviation was significantly more prevalent among anisometropic patients (36.5% vs. 3.25%, *P* < 0.001) and exotropia was the common type of deviation. Anisohyperopia and anisomyopia were the most common refractive errors under low and high anisometropia categories, respectively. Simultaneous manifestation of amblyopia and strabismus were observed in 30.6% of anisometropic cases, while only 0.7% of subjects with isometropia had a similar status (*P* < 0.001).

**Conclusion:**

High rates of amblyopia and strabismus in anisometropic subjects, especially with higher degrees of anomaly, indicate the necessity of early visual acuity and refractive error screening to improve detection and enhance the outcomes of treatment.

##  INTRODUCTION

Anisometropia is a leading cause of amblyopia and binocular dysfunction.^[1]^ According to the Pediatric Eye Disease Study, amblyopia can be identified in > 60% of children with anisometropia of 2.00D or more. This threshold is lower for hyperopic spherical equivalent (SE) difference of 1.50D and higher for myopia (SE difference of 3.00D).^[2]^ High anisometropia is considered when SE difference exceeds 3.00D between the two eyes, which causes aniseikonia, amblyopia, confusion, strabismus, and diplopia.^[3,4]^ The prevalence of anisometropia (SE difference ≥ 1.00D) was reported to be 5.3% in a population-based study conducted on 23,114 individuals,^[5]^ and 11.2% in Irish children aged 12–13 years.^[6]^ Most anisometropic patients do not tolerate glasses particularly when their fellow eye has good visual acuity. Contact lens correction would reduce aniseikonia and increase tolerability of optical correction, however, the drawbacks include the risk of infection, foreign body sensation, fitting and handling problems, and high cost limiting its use especially in children.^[7,8,9]^ Keratorefractive surgery improves visual acuity and stereopsis but entails side effects of corneal haze, ectasia, and recurrence documented in case reports; however, randomized controlled trials are very rare in this regard.^[3,4]^ In the present study, we compare the ocular and demographic characteristics of subjects with low and high levels of anisometropia as compared to non-asnisometropic individuals and the management at the eye centers affiliated to Shahid Beheshti University of Medical Sciences, Tehran, Iran between 2019 and 2020.

##  METHODS

**Figure 1:**
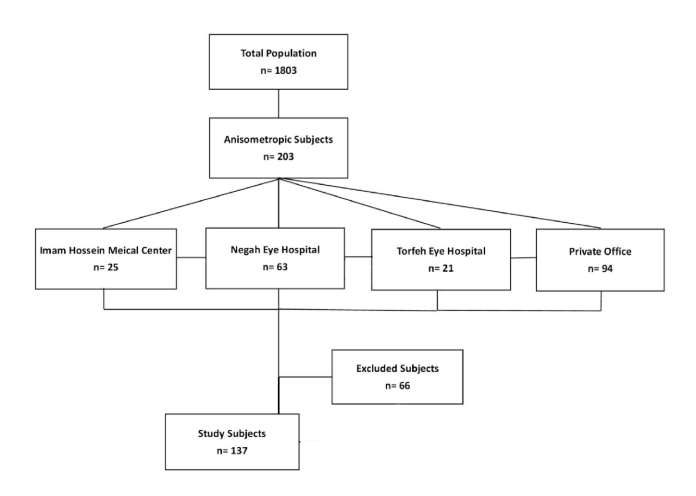
Workflow of the present study. n, number.

This cross-sectional study was conducted on a total of 1803 subjects with an age range of 1 to 30 years examined at strabismus clinics of eye centers affiliated to Shahid Beheshti University between January 2019 and December 2020. Of these, 203 (11.2%) cases had anisometropia with a minimum spherical or cylindrical difference of 1.50D between the two eyes. The study population were selected from Negah Eye Hospital (n = 63), Imam Hossein Medical Center (n = 25), Torfeh hospital (n = 21), and a private ophthalmology office (n = 94) (ZR) [Figure 1]. The remaining 1600 (88.7%) individuals with no difference in spherical or cylindrical refractive errors served as controls.

Before starting the study, skilled optometrists in the aforementioned centers passed a 2-hr training course for coordination in optometric examinations and filling out the forms (Cronbach's alpha = 0.76). Cases with history of infantile cataracts and ocular surgeries were excluded from the final analysis.

All study stages adhered to the Declaration of Helsinki and were approved by the Ethics Committee of the Ophthalmic Research Center affiliated to Shahid Beheshti University of Medical Sciences (IR.SBMU.ORC.REC.1399.017). Patients or their parents were asked to sign the informed consent form prior to examination. Demographic questions including age, sex, past medical history of the patient and his/her family were recorded in the questionnaire. Afterward, best corrected visual acuity (BCVA) was measured by linear Snellen visual acuity E-charts at far distance (6 m) under daylight conditions. In younger children, we asked their parents to teach E-game to them before E-chart testing. Most of them were able to respond to VA testing; otherwise, their findings were considered as not available. A diagnosis of amblyopia was made if BCVA was worse than 0.3 LogMAR in either eye or there was a difference in BCVA of two or more lines between the two eyes. Cycloplegic refraction was measured using an autorefractometer or retinoscope in younger and uncooperative children, 30–45 minutes after instillation of one drop of tropicamide 1% and cyclopentolate 1% 5 minutes apart and refraction was recorded in minus cylindrical form. Cases with a combination of spherical and cylindrical refractive errors in each eye were considered as hyperopia-astigmatism or myopia-astigmatism. A minimum spherical or cylindrical difference of 1.50D or 3.00D between the two eyes was defined as low or high anisometropia, respectively. Anisohyperopia, anisomyopia, or anisoastigmatism were considered if the SE difference between the two eyes was positive or negative or referred to only the cylindrical refractive error of the two eyes.^[1]^ Those with no difference in spherical or cylindrical refractive errors were considered as controls.

Extraocular muscle function was assessed by the ocular motility test at nine cardinal gazes and it was recorded from +4 to –4 for maximum to minimum muscle function, respectively. Ocular deviation was measured by Krimsky in young or non-cooperative cases, and alternative prism cover test at both far (6 m) and near (33 cm) distances. Anterior and posterior ocular segments were examined by slit-lamp biomicroscopy and indirect ophthalmoscopy, respectively.

Based on the amount of the refractive errors and the threshold of the patients' tolerance for contact lenses (if glasses were rejected), contact lens wear was suggested especially when ansimetropia was high.

### Statistical Analysis

Mean, standard deviation, median and range, frequency, and percentage were used to present data. Chi-Square test was applied to evaluate the difference between fellow eyes for qualitative variables such as anisometropia. Generalized estimating equations (GEE) was used to evaluate the possible correlations of results in fellow eyes, if necessary. All statistical analyses were performed using the SPSS (IBM Corp. Released 2017. IBM SPSS Statistics for Windows, Version 25.0. Armonk, NY: IBM Corp.). *P*-values < 0.05 were considered as statistically significant.

**Figure 2:**
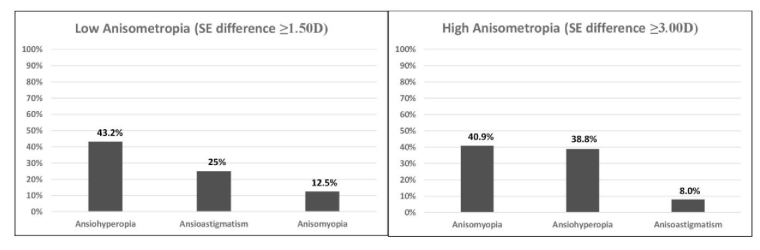
The frequency of amblyopia in patients with low and high anisometropia in different types of refractive errors. SE, spherical equivalent; D, diopter

**Table 1:** Epidemiologic characteristics of all the study subjects


**Factors**	**Level**	**Anisometropia (** ***n*** ** = 137)**	**Non-anisometropia (** ***n*** ** = 1600)**	***P*** **-value**
Sex (%)	Male	59 (43.1%)	828 (51.8%)	0.051
	Female	78 (56.9%)	772 (48.3%)	
Age (yrs)	Mean ± SD	10.25 ± 8.41	9.2 ± 1.7	0.133
	Median (Range)	7 (1 to 30)	9 (6 to 13)	
PG in Family (%)	No	75 (54.7%)	1106 (69.1%)	0.0005
	Yes	62 (45.3%)	494 (30.9%)	
	
	
PG, present glasses; yrs, years; SD, standard deviation; n, number							

**Table 2:** Clinical findings of our study subjects


**Factors**	**Level**	**Anisometropia (** ***n*** ** = 137)**	**Non-anisometropia (** ***n*** ** = 1600)**	***P*** **-value**
Amblyopia (%)	Yes	Unilateral	58 (42.3%)	23 (1.4%)	< 0.001
	Bilateral	56 (40.9%)	14 (0.9%)	
	No	23 (16.8%)	1563 (97.7%)	
BCVA (LogMAR)	Amblyopic eye	Mean ± SD	0.34 ± 0.46	0.37 ± 0.15	0.468
	Median (Range)	0.15 (0.0 to 2.7)	0.3 (0.3 to 0.9)	
	Non-Amblyopic eye	Mean ± SD	0.06 ± 0.17	0.01 ± 0.01	0.001
	Median (Range)	0 (0.0 to 1.09)	0.1 (0.1 to 0.3)	
	*P*-within	< 0.001	< 0.001	
Ocular alignment at Far (%)	No strabismus	87 (63.5%)	1548 (96.75%)	< 0.001
	Yes	50 (36.5%)	52 (3.25%)	
Type of strabismus (%)	ET	21 (15.3%)	21 (1.3%)	0.957
	XT	27 (19.7%)	28 (1.75%)	
	HT	1 (0.7%)	1 (0.06%)	
	HOT	1 (0.7%)	2 (0.12%)	
RE (%)	Hyperopic astigmatism	69 (50.4%)	1382 (86.3%)	< 0.001
	Myopic astigmatism	36 (26.3%)	8 (0.5%)	
	H or M or As alone	32 (23.3%)	210 (13.2%)	
Type of treatment (%)	Patch	129 (58.6%)	
	Glasses	124 (90.5%)	
	Contact lens	12 (8.8%)	
	Photorefractive surgery	1 (0.7%)	
	
	
BCVA, best corrected visual acuity; LogMAR, logarithm minimum angle of resolution; SD, standard deviation; ET, esotropia; XT, exotropia; HT, hypertropia; HOT, hypotropia; RE, refractive error H, hyperopia; M, myopia; AS, astigmatism					

**Table 3:** The percentage of strabismus and amblyopia in patients with anisometropia and non-anisometropia

**Factors**	orange**Anisometropia**										orange**Total Anisometropia (1)**			orange**Non-Anisometropia (2)**			***P*** **-value**	***P*** ** (1),(2)**
	orange**Low (1.50 ≤ Dif. SE ≤ 3.00D)**			***P*** **-value**	orange**High (Dif. SE > 3.00D)**			***P*** **-value**	**Non-Amb.**	**Amb.**	***P*** **-value**	**Non-Amb.**	**Amb.**	
	**Non-Amb.**	**Amb.**		**Non-Amb.**	**Amb.**						
Deviation at far	Horizontal	5 (18.5%)	22 (81.5%)	< 0.001	2 (9.5%)	19 (90.5%)	0.025	7 (14.6%)	41 (85.4%)	0.415	9 (18.4%)	40 (81.6%)	< 0.001	< 0.001
	Vertical	0 (0.0%)	1 (100.0%)		1 (100.0%)	0 (0.0%)		1 (50%)	1 (50%)		1 (33.3%)	2 (66.7%)	
	No deviation	12 (20.0%)	48 (80.0%)		3 (11.1%)	24 (88.9%)		15 (17.2%)	72 (82.8%)		1522 (98.3%)	26 (1.7%)	
	Total	17 (19%)	71 (81%)		6 (12.2%)	43 (87.8%)		23 (16.7%)	114 (83.3%)		1563 (97.64%)	37 (2.36%)	
	
	
white<bcol>22</ecol>SE, spherical equivalent; Dif., difference; Amb., amblyopia; D, diopter

##  RESULTS

In this cross-sectional study, a total of 1803 patients were examined. Overall, 203 patients were anisometropic (11.2%) with a mean age of 13.8 ± 15.8 years. History of previous ocular surgery was reported in 66 (32%) cases, after exclusion of which, data from 137 patients were analyzed. The mean age was 10.25 ± 8.41 years in anisometropic and 9.2 ± 1.7 years in non-anisometropic patients (*P* = 0.133) [Table 1]. Amblyopia was detected in 83% of the anisometropic subjects and the mean BCVA in these eyes was 0.34 ± 0.46 LogMAR versus 0.06 ± 0.17 LogMAR in non-amblyopic fellow eyes (*P* < 0.001). The rate of amblyopia was 2.3% in non-anisometropic control subjects; vision in the amblyopic eyes of the control group was comparable to cases but BCVA in their fellow eyes (0.01 ± 0.01 LogMAR) was higher than non-amblyopic eyes in the case group [Table 2]. The prevalence of amblyopia was higher in subjects with high anisometropia as compared to low anisometropia (87.8% vs. 81%). Amblyopia was not present in 17 (19.3%) low anisometropic patients (including 11 [64%] aniso-hyperopic and 6 [36%] aniso-myopic cases]) and 6 (12.3%) high anisometropic patients (including 2 [33%] aniso-hyperopic and 4 [67% aniso-myopic cases) [Table 3; Figure 2]. On the other side, 2.3% amblyopia were seen in non-anisometropic cases.

Strabismus was present in 36.5% and 3.25% of anisometropic and non-anisometropic subjects, respectively. Most of them were horizontally deviated and exotropia was the common type of deviation in both study groups [Table 2]. Amblyopia and strabismus were simultaneously present in 23 (30.6%) and 11 (0.7%) anisometropic and non-anisometropic cases, respectively (*P* < 0.001) [Table 3]. Aniso-hyperopia and younger age in low (*P* = 0.047) and aniso-myopia and horizontal deviation in high anisometropia (*P* = 0.046) were the most influential risk factors for amblyopia in anisometropic patients. Anisometropia was managed by glasses in nearly all patients and the dominant eye was suggested to receive part-time patching in subjects under the age of 12 years. After the exclusion of unilateral congenital cataract cases who had to wear contact lens postoperatively, only 12 (24.5%) patients with high anisometropia wore contact lenses. One 25-year-old patient had anisometropic amblyopia with esotropia and history of photorefractive keratectomy and strabismus surgery at the age of 13 years, in separate sessions but did not achieve BCVA improvement in her amblyopic eye (BCVA = 1.0 LogMAR).

##  DISCUSSION

Anisometropia was present in 203 individuals out of 1803 study subjects (11.2%) who were referred to our clinics. After excluding cases with history of ocular surgery, the results of 137 anisometropic patients were finally analyzed and compared with non-anisometropic cases.

Appropriate glasses were prescribed for all patients with refractive errors. However, glasses tolerance is not easy especially when the fellow eye has good visual acuity. If glasses are rejected, contact lenses are suggested especially with high ansimetropia.^[14]^ Contact lens decreases aniseikonia, and provide better tolerance to optical correction, however, they involve fitting, handling, changing, and cost problems.^[14]^ If none of the aforementioned corrections are effective, keratorefractive surgery may rarely be suggested as the last solution for the management of anisometropia in children.^[3,4][9]^ Patching or penalization of the dominant eye is suggested as adjunctive treatment for amblyopic patients under the age of 12 years.

Based on our results, amblyopia was present in 83% of anisometropic subjects with a mean BCVA of 0.34 ± 0.46 LogMAR in amblyopic eyes versus 0.06 ± 0.17 LogMAR in non-amblyopic fellow eyes (*P* < 0.001). Among the non-inasometropic control group amblyopia was present in 2.3% of subjects with similar vision in amblyopic eyes but higher BCVA 0.01 ± 0.01 in non-amblyopic fellow eyes. The prevalence of amblyopia was greater with high anisometropia as compared to low anisometropia (87.8% vs. 81%) as expected. Surprisingly, 17 (19%) low anisometropic and 6 (12.2%) high anisometropic patients did not show any amblyopia. Strabismus was found in 36.5% and 3.25% of cases with anisometropia and non-anisometropia, respectively. Most of them were horizontally deviated and exotropia was the common type of deviation in both groups.

In the systematic review and meta-analysis conducted by Hashemi et al, the most common cause of amblyopia was anisometropia (61.5%).^[15]^ In the studies performed in Iran, China, and Bulgaria, the rates of amblyopic anisometropia were 46%, 40%, and 59%, respectively and the condition was the leading cause of amblyopia.^[16,17,18]^ The criteria for anisometropia in all of these studies were between 1.00 and 2.00D, showing that 40% to 60% of amblyopia was due to anisometropia.^[15][16,17,18]^

In our study, 80% and 87% of cases with low and high anisometropia were amblyopic as compared to 2.3% in non-anisometropic cases. This higher percentage of amblyopia secondary to anisometropia in the current study could be due to the referral nature of our cases in comparison to population-based studies.

Surprisingly, 19% of the patients with low anisometropia and 12.2% of patients with high anisometropia in our study did not show any amblyopia possibly due to detection and management of anisometropia at a young age. Furthermore, it was found that 11 (0.7%) of non-anisometropic individuals suffered from amblyopia, which could have resulted from the amount of anisometropia and the other amblyopic risk factors such as strabismus.

After exclusion of patients with unilateral congenital cataract, all highly anisometropic patients were suggested to wear contact lenses; however, only a minority of them (*n* = 12, 24.5%) accepted this recommendation possibly due to contact lens handling problems and high cost in addition to other issues such as changes in refractive error or contact lens damage. Wang et al^[19]^ reported that the rigid gas permeable (RGP) contact lens could be a safe and effective modality for children. In their study, myopic children wore RGP contact lens and were followed for five years.^[19]^

In our study, one patient had a history of keratorefractive surgery at the age of 13 years for correction of anisometropia. Although she had a history of photorefractive keratectomy and strabismus surgery, visual acuity in her amblyopic eye did not change (BCVA = 1.0 LogMAR) due to late management.

The low number of anisometropic patients undergoing keratorefractive surgery in childhood in the current study shows the low tendency of our surgeons and parents to accept these types of treatment even when other methods have not been effective. This belief may be changed appropriately in future.

Although keratorefractive surgery can improve VA and stereopsis in anisometropic children, it is not a perfect type of treatment and has complications such as regression and corneal haze especially in high refractive errors, indicating the need for long-term follow-up of these children.^[3,6,9,20,21,22]^ Hyperopia at younger age in anisometropic group and myopia and strabismus in non-anisometropic group are the risk factors which necessitate paying attention to the age and type of the refractive error in these patients.

Classification of anisometropia into low and high levels to further clarify their ocular effects and determining the risk factors of amblyopia in each group are the strengths and the small number of contact lens wearers and cases undergoing keratorefractive surgery are the limitations of this study.

In summary, high rates of amblyopia and strabismus in patients with anisometropia, especially in higher degrees, underscore the importance of early screening for visual acuity and cycloplegic refraction to detect and treat amblyopia and anisometropia as soon as possible. In addition, we may suggest paying attention to age and type of refractive error as risk factors for amblyopia in anisometropic patients.

##  Financial Support and Sponsorship

None.

##  Conflicts of Interest

None.

## References

[B1] Rajavi Z, Sabbaghi H, Baghini AS, Yaseri M, Moein H, Akbarian S, et al (2015). Prevalence of amblyopia and refractive errors among primary school children. J Ophthalmic Vis Res.

[B2] Wang B, Naidu RK, Qu X (2018). The use of rigid gas permeable contact lenses in children with myopic amblyopia: A case series. Cont Lens Anterior Eye.

[B3] Zhang J, Yu KM (2017). Femtosecond laser corneal refractive surgery for the correction of high myopic anisometropic amblyopia in juveniles. Int J Ophthalmol.

[B4] Kraus CL, Culican SM (2018). New advances in amblyopia therapy II: Refractive therapies. Br J Ophthalmol.

[B5] Lee CW, Fang SY, Tsai DC, Huang N, Hsu CC, Chen SY, et al (2017). Prevalence and association of refractive anisometropia with near work habits among young schoolchildren: The evidence from a population-based study. PLoS One.

[B6] Harrington S, Breslin K, O’Dwyer V, Saunders K (2019). Comparison of amblyopia in school children in Ireland and Northern Ireland: A population-based observational cross-sectional analysis of a treatable childhood visual deficit. BMJ Open.

[B7] McNeill S, Bobier WR (2017). The correction of static and dynamic aniseikonia with spectacles and contact lenses. Clin Exp Optom.

[B8] Hepschke JL, Ung L, Cabrera-Aguas M, Ross C, Kumar N, Lahra MM, et al (2020). Pediatric microbial keratitis: Experience from tertiary referral centers in New South Wales, Australia. Pediatr Infect Dis J.

[B9] Kulikova IL, Pashtaev NP, Batkov YN, Pikusova SM, Terent’eva AE (2020). Femtosecond laser-assisted lasik in children with hyperopia and anisometropic amblyopia: 7 years of follow-up. J Refract Surg.

[B10] Eissa SA (2017). Management of pseudophakic myopic anisometropic amblyopia with piggyback Visian® implantable collamer lens. Acta Ophthalmol.

[B11] Vasavada V, Srivastava S, Vasavada SA, Sudhalkar A, Vasavada AR, Vasavada VA (2018). Safety and efficacy of a new phakic posterior chamber IOL for correction of myopia: 3 years of follow-up. J Refract Surg.

[B12] Yildirim Y, Cakmak S, Sucu ME, Kepez Yildiz B, Kirgiz A, Akbas YB, et al (2020). Comparative study of small-incision lenticule extraction and phakic intraocular lens implantation for the correction of high myopia: 6-year results. J Cataract Refract Surg.

[B13] Sucu ME, Cakmak S, Yildirim Y, Yildiz BK, Yalçınkaya G, Beşek NK, et al (2020). Explantation of phakic intraocular lenses: causes and outcomes. Int Ophthalmol.

[B14] Krarup TG, Nisted I, Christensen U, Kiilgaard JF, la Cour M (2020). The tolerance of anisometropia. Acta Ophthalmol.

[B15] Hashemi H, Pakzad R, Yekta A, Bostamzad P, Aghamirsalim M, Sardari S, et al (2018). Global and regional estimates of prevalence of amblyopia: A systematic review and meta-analysis. Strabismus.

[B16] Faghihi M, Hashemi H, Nabovati P, Saatchi M, Yekta A, Rafati S, et al (2017). The prevalence of amblyopia and its determinants in a population-based study. Strabismus.

[B17] Li YP, Zhou MW, Forster SH, Chen SY, Qi X, Zhang HM, et al (2019). Prevalence of amblyopia among preschool children in central south China. Int J Ophthalmol.

[B18] Dikova SP, Dragoev SA, Chernodrinska VS (2018). Prevalence of amblyopia in Bulgaria. Strabismus.

[B19] Wang B, Naidu RK, Qu X (2018). The use of rigid gas permeable contact lenses in children with myopic amblyopia: A case series. Cont Lens Anterior Eye.

[B20] Tychsen L (2008). Refractive surgery for children: Excimer laser, phakic intraocular lens, and clear lens extraction. Curr Opin Ophthalmol.

[B21] Dvali ML, Tsintsadze NA, Mirtskhulava SI

[B22] Astle WF, Fawcett SL, Huang PT, Alewenah O, Ingram A (2008). Long-term outcomes of photorefractive keratectomy and laser-assisted subepithelial keratectomy in children. J Cataract Refract Surg.

